# Effects of Pain Relief Through Minimal Exercise Intervention in a Rat Model of Neuropathic Pain

**DOI:** 10.7759/cureus.62897

**Published:** 2024-06-22

**Authors:** Megumi Sumizono, Yushin Yoshizato, Takaki Imai, Akira Tani, Kazuki Nakanishi, Nao Nojima, Shogo Kakimoto, Harutoshi Sakakima

**Affiliations:** 1 Rehabilitation, Kyushu University of Nursing and Social Welfare, Tamana, JPN; 2 Physical Therapy, School of Health Sciences, Kagoshima University, Kagoshima, JPN

**Keywords:** beta-endorphin/met-enkephalin, traf6, ccr2, minimal motor intervention, neuropathic pain

## Abstract

We aimed to minimize the frequency of exercise intervention and test the efficacy of pain relief. We also investigated the mechanism of neuropathic pain to determine the best frequency of pain relief for neuropathic pain. The chronic constriction injury (CCI) rat model was randomly divided into three groups: exercise (Ex), No-Ex, and normal. The treadmill exercise intervention was administered, and the 50% withdrawal threshold was assessed using the Von Frey Test. Ionized calcium-binding adaptor molecule 1 (IBA1), glial fibrillary acidic protein (GFAP), brain-derived neurotrophic factor (BDNF), C-C chemokine receptor type 2 (CCR2), and tumor necrosis factor receptor-associated factor 6 (TRAF6) activation was determined through immunohistochemistry. In the brain, we examined the increased expression of β-endorphin/met-enkephalin in the gray matter of the midbrain aqueduct. Co-expression of CCR2, IBA1, and Neu-N was observed in the spinal cord dorsal horn by immunofluorescence staining. The 50% pain response threshold was significantly lower in the Ex group than in the No-Ex group at five weeks post-CCI, indicating a high analgesic effect. In the dorsal horn of the spinal cord, IBA1 and GFAP were significantly decreased in the Ex group than in the No-Ex group at five weeks post-CCI. However, no significant difference in activation of BDNF, CCR2, and TRAF6 was observed. In the midbrain, the Ex group showed a significant increase compared to the No-Ex group. In summary, our results suggest that in minimal-exercise intervention, neuropathic pain relief is achieved by activation of the descending pain inhibitory system in the midbrain.

## Introduction

Neuropathic pain arises from central or peripheral nerve damage, activating microglia and astrocytes in the dorsal horn of the spinal cord and causing pain through inflammatory substance production and abnormal neuronal function [1-3]. Additionally, developing an effective treatment is challenging.

Chronic pain is a severe social issue in Japan due to its aging population, and its prevalence is approximately 40% [4]. The World Health Organization launched the Decade of Bone and Joint Disorders in 2000, aiming to improve people’s health-related quality of life with associated complications and social costs. In 2006, the Japanese Orthopedic Association proposed the concept of locomotive syndrome (LS) to reduce the number of older adults requiring care [5]. In Japan, LS-related diseases are categorized as musculoskeletal diseases, and a prescribed number of points is calculated as the musculoskeletal rehabilitation fee. The calculation’s upper limit is defined as 150 days. Patients who have exceeded the standard calculation days are limited to only 13 units of the prescribed fee for locomotor rehabilitation monthly, placing significant restrictions on rehabilitation. Exercise prevented pain onset and eliminated pain after injury, and 58/64 (90%) articles published on exercise-induced analgesia showed a positive effect of exercise with only one activity [6]. These studies focused on immediate effects rather than ongoing interventions. Therefore, the effects of minimal exercise interventions require regular evaluation.

Microglia become activated after nerve injury. C-C motif chemokine receptor 2 (CCR2) is present in activated microglia. C-C chemokine ligand 2 (CCL2)-CCR2 binding modulates central sensitization via the N-methyl-D-aspartate receptor. The CCL2/CCR2-dependent mechanism plays an essential role in neuropathic pain development [7]. Chemokines induce glial cell activation, promote excitatory synaptic transmission in spinal cord neurons, and exaggerate central sensitization. Moreover, this depends on the differential distribution of ligand receptors in neurons and glial cells [8]. Astrocytes are primarily involved in the subsequent stages of neuropathic pain; tumor necrosis factor (TNF) receptor-associated factor 6 (TRAF6) is expressed on astrocytes associated with TNF-α and interleukin (IL)-1β signaling and activates the c-Jun N-terminal kinase (JNK)/CCL2 pathway in astrocytes, thereby perpetuating neuropathic pain [9,10].

Many reports exist on the effectiveness of pharmacological interventions in neuropathic pain treatment; however, only a few studies have reported exercise intervention effectiveness, and the pain reduction mechanism remains unclear [11,12]. Opioid therapy for neuropathic pain is usually discouraged due to concerns about its ineffectiveness, the potential for tolerance, the risk of addiction, and several side effects. However, potent opioid analgesics, such as morphine, fentanyl, and oxycodone, are effective in managing steady-state pain, paroxysmal spontaneous pain, and allodynia common in postherpetic neuralgia [13]. Endogenous opioids have been reported to increase after 5-6 and 8 weeks of exercise training, reverse naloxone-mediated sensory hypersensitivity, and elevate brainstem opioid content, subsequently alleviating pain following five weeks of exercise training [14,15].

Our previous study examined the effects of different exercise frequencies in a neuropathic pain rat model. We reported that neuropathic pain was alleviated by glial cell activation, brain-derived neurotrophic factor (BDNF) suppression in the ipsilateral spinal dorsal horn, and endogenous opioid system regulation. However, the exercise intervention frequency varied. We also reported the analgesic effects of exercise intervention by activating the descending pain inhibitory system through an increase in the endogenous opioid β-endorphin/met-enkephalin in the gray matter of the midbrain aqueduct [16]. We revealed the relationship between neuropathic pain-induced expression of spinal glial cells, CCR2, and TRAF6; the dynamics of doublecortin and Prospero homeobox protein 1 in the hippocampal dentate gyrus; and the effects of regular exercise therapy. The results showed that TRAF6 is a glial cell therapeutic target and a target of interest in maintaining neuropathic pain [17]. Ongoing mechanistic research will facilitate intervention target development for chronic pain treatment, and we believe that safer and more effective pain relief will be less burdensome for patients with neuropathic pain. This study minimized the frequency of exercise interventions and verified the pain relief effect of neuropathic pain to verify the best frequency of pain relief for neuropathic pain.

## Materials and methods

Animals

Male Sprague-Dawley rats (eight-week-old, n = 16) weighing 254.2 ± 5.4 g (mean ± standard error) were used in this experiment. Rats were subjected to a 12-hour light/dark cycle in a temperature-controlled room (23.0 ± 1.0°C) with ad libitum access to solid feed and water. The experiments were conducted with a minimum number of animals to collect the same information with few animals or the maximum information with few animals kept for an extended period. This study was approved by the Kagoshima University Faculty of Medicine Animal Experimentation Committee (approval number: M20002). The protocols were in accordance with the Classification of Biomedical Experimental Processes Based on Ethical Considerations for Nonhuman Species (Consensus Recommendations for Effective Institutional Animal Experimentation).

CCI model

Rats were anesthetized by intraperitoneal injection of a 4% chloral hydrate solution (10 mL/kg). The skin covering the right thigh was incised and carefully dissected to avoid sciatic nerve injury. The CCI is a slight modification of the method used by Bennett and Xie [18]. Briefly, four ligating sutures (4-0 silk) were loosely tied around the right sciatic nerve, approximately 1 mm apart. The length of the nerve affected by ligature was approximately 6 mm. The incision was closed with 4-0 sutures, and the animal was returned to the cage for recovery. The contralateral hind limb was left untreated and used as a control for the mechanical sensitivity of the hind limb.

Regular treadmill exercise and experimental group

The exercise was performed on a treadmill (MK-680, Muromachi Kikai Co., Ltd., Tokyo, Japan), and all rats were subjected to environmental adaptation at a speed of 20 m/min for 15 min and 1.0 A voltage for three days. After environmental adaptation, the rats were randomly divided into three groups: the CCI exercise group (Ex, n = 4), the CCI non-exercise group (No-Ex, n = 4), and the normal group (normal, n = 4). Exercise consisted of running on a treadmill at a rate of 20 m/min for one day weekly. The exercise was performed after free-cage recovery for one or two days after CCI. The treadmill exercise program was initiated 2-3 days after CCI at a speed of 20 m/min for 15 min. Exercise duration was increased to 30 min each day after exercise and maintained until five weeks after CCI. The working rate of the rats at this training rate was approximately 55% of their maximal oxygen consumption [19,20]. Body weight was measured periodically to monitor stress induced in the rats by treadmill exercise. None of the animals was excluded from the study.

Change in mechanical sensitivity of the hind paw

Both hindlimbs evaluated mechanical sensitivity as withdrawal response frequency using Von Frey filaments (Muromachi Kikai Co., Ltd., Japan). Measurements were taken before CCI and at one, two, three, four, and five weeks after CCI (between 1 pm and 5 pm). The rats were placed in individual clear plastic cages on wire mesh grates with full access to the ventral aspect of the hindlimb. Measurements were taken before the exercise intervention, and the animals were acclimated to the experimental environment (room and apparatus) for at least 20 min. A logarithmic series of 11 filaments (0.41-28.84 g) was pressed vertically onto the plantar surface of the hindfoot until the filaments were bent. The test was initiated with 3.63 g filaments. The top-down method was used in this study. Tactile stimuli that elicited a 50% probability of hind limb withdrawal response (50% withdrawal threshold) were calculated using the following formula [21]

Histology and immunohistochemistry

Rats were sacrificed, and histological and immunohistochemical analyses were performed after five weeks (Ex: n = 4, No-Ex: n = 4). The intact group of rats (n = 4) was used as a normal control group for histological and immunohistochemical analyses at the end of the experiment. Every effort was made to reduce the number of animals used in the study.

Rats were first administered 4% chloral hydrate (10 mL/kg, intraperitoneal overdose) before perfusion and fixed reflux-fixed in heparin saline, 4% paraformaldehyde 0.1 M phosphate buffer (pH 7.4) via the heart. The lumbar portions of the spinal cord and midbrain were removed and fixed overnight at 4°C. After fixation, the tissues were dehydrated and embedded in paraffin, and 5 μm-thick sections were stained with hematoxylin and eosin (HE) to observe histological changes. In addition, immunohistochemical changes after CCI were analyzed.

After deparaffinization, the sections were immersed in 3% H₂O₂ solution for 10 min to inactivate endogenous peroxidase. After three five-min washes with phosphate-buffered saline (PBS, pH 7.6), the sections were incubated with 10% skim milk in PBS to block specific sites. The sections were rinsed three times for 5 min each in PBS and incubated individually at 4°C with one of the following antibodies: rabbit anti-ionized calcium-binding adaptor molecule 1 (IBA1) antibody (1:2000, rabbit polyclonal, Wako Pure Chemical Industries, Osaka, Japan), a marker for microglia; rabbit anti-glial fibrillary acidic protein (GFAP) antibody (1:1000, rabbit polyclonal, Shima Research Institute, Tokyo, Japan), a marker for astrocytes; goat anti-CCR2 antibody (1:1500, goat polyclonal (1:600, mouse monoclonal, Proteintech Group, Inc., USA), a marker for TRAF6; anti-BDNF antibody, a marker for cranial nerve-derived neurotrophic factor (BDNF) (1:600, mouse monoclonal, Proteintech Group, Inc., USA); and anti-BDNF antibody (1:200, rabbit polyclonal, Bioss Antibodies Inc.); and anti-β-endorphin/met-enkephalin antibody, a marker for endogenous opioids (1:50; Santa Cruz Biotechnology, Inc.). After incubation with primary antibodies, IBA1, GFAP, TRAF6, and BDNF, the sections were rinsed with PBS three times for 5 min each. The sections were then incubated with goat anti-rabbit immunoglobulin G (IgG) or anti-mouse IgG conjugated to a peroxidase-labeled dextran polymer (EnVision; Dako, USA) for 60 min. The incubated sections were subjected to three 5-min PBS washes, followed by visualization of section immunoreactivity using diaminobenzidine (DAB) peroxide. For CCR2, anti-β-endorphin/met-enkephalin antibodies, the ABC kit (Vector Laboratories, Burlingame, USA) was used according to the manufacturer's instructions. After three 5-min PBS washes, the immunoreactivity of the sections was visualized using DAB peroxide.

IBA antibody was co-localized (1:500) with anti-mouse CCR2 antibody (1:500) and anti-mouse Neu-N (a marker of neurons) antibody (1:100, rabbit mAb, Cell Signaling Technology, Inc., Japan) with anti-CCR2 antibody staining. After incubation with the primary antibody and PBS washing, sections were incubated with Alexa Fluor 488-conjugated goat anti-rabbit IgG (1:200) and Alexa Fluor 546-conjugated goat anti-mouse IgG (1:200) for 60 min. The sections were counterstained with PBS and 4′ 6-diamidino-2-phenylindole for 10 min. Finally, the samples were mounted in an aqueous mounting medium. Immunofluorescence staining was observed under a fluorescence microscope (EVOS f1; AMG, Mill Creek, USA).

Quantitative immunostaining analysis

A digital camera for light microscopy (DP21, Olympus Optical Co., Tokyo, Japan) was used to capture stained sections of the lumbar spine and cerebrum at 10× magnification. The ratio of the immunoreactivity of anti-IBA1, GFAP, CCR2, and TRAF6 containing laminae I-III in the dorsal horn of the spinal cord was quantitatively measured using ImageJ software (NIH, USA). Immunostained areas were measured in the ipsilateral and contralateral dorsal horns of the spinal cord. Quantitative analysis of each immunostained section was performed by two or three individuals blinded to the treatment group. This method of analysis was used because it allowed the quantification of the percentage of immunostained areas.

Statistical analysis

Data were expressed as the mean ± standard error. A two-way analysis of variance (group × time) was used for the analysis of the time course of the 50% withdrawal threshold, followed by the Bonferroni test for multiple comparisons. Where appropriate, a one-way analysis of variance followed by Tukey’s post-hoc test was used to analyze the proportion of immunostained areas. Statistical significance was set at p < 0.05, and the data were analyzed using IBM SPSS Statistics for Windows, Version 25 (Released 2017; IBM Corp., Armonk, New York, USA). Only graphing was performed with GraphPad Prism 9.3.1 (471) (GraphPad Holdings, LLC, San Diego, USA).

## Results

Reduction of sensory hypersensitivity after chronic constriction injury (CCI) through exercise

**Figure 1:**
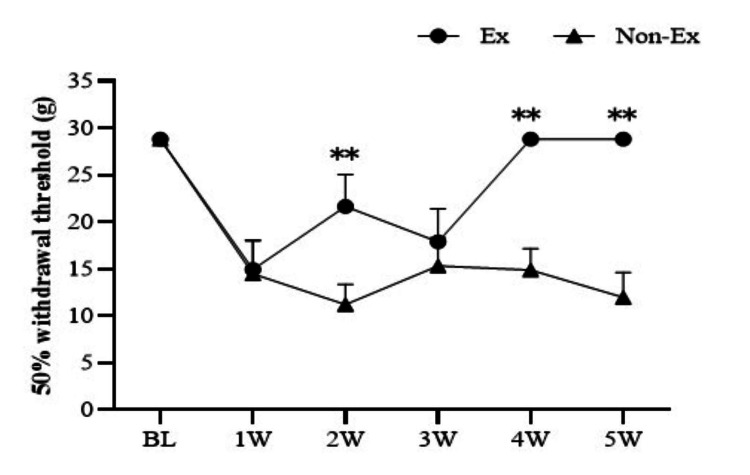
Ex and No-Ex groups showed nerve injury causing hyperalgesia. The chronic strangulation injury (CCI) and non-exercise group (i.e., No-Ex group) showed a trend toward recovery with maximum hyperalgesia at two weeks but an acute exacerbation at five weeks after CCI. Data expressed as mean ± SE; * p < 0.05, ** p < 0.01 compared to the No-Ex group. Ex: exercise

The 50% withdrawal threshold (g) was evaluated over time before CCI (n = 4, 4), one week (n = 4, 4), two weeks (n = 4, 4), three weeks (n = 4, 4), four weeks (n = 4, 4), and five weeks (n = 4, 4) in the Ex and No-Ex groups using the Von Frey test. The cutoff value was set at 28.84 g (Figure 1).

The Ex group showed a trend toward improvement in the pain-response thresholds compared to the No-Ex group. The Ex group showed significantly greater improvement at two weeks post-CCI (p < 0.01) but showed a temporary exacerbation at three weeks post-CCI. On the other hand, the No-Ex group showed a drop in pain threshold and exacerbation of pain.

Exercise intervention suppresses IBA1, GFAP, and BDNF in the dorsal horn of the spinal cord at five weeks post-CCI

**Figure 2:**
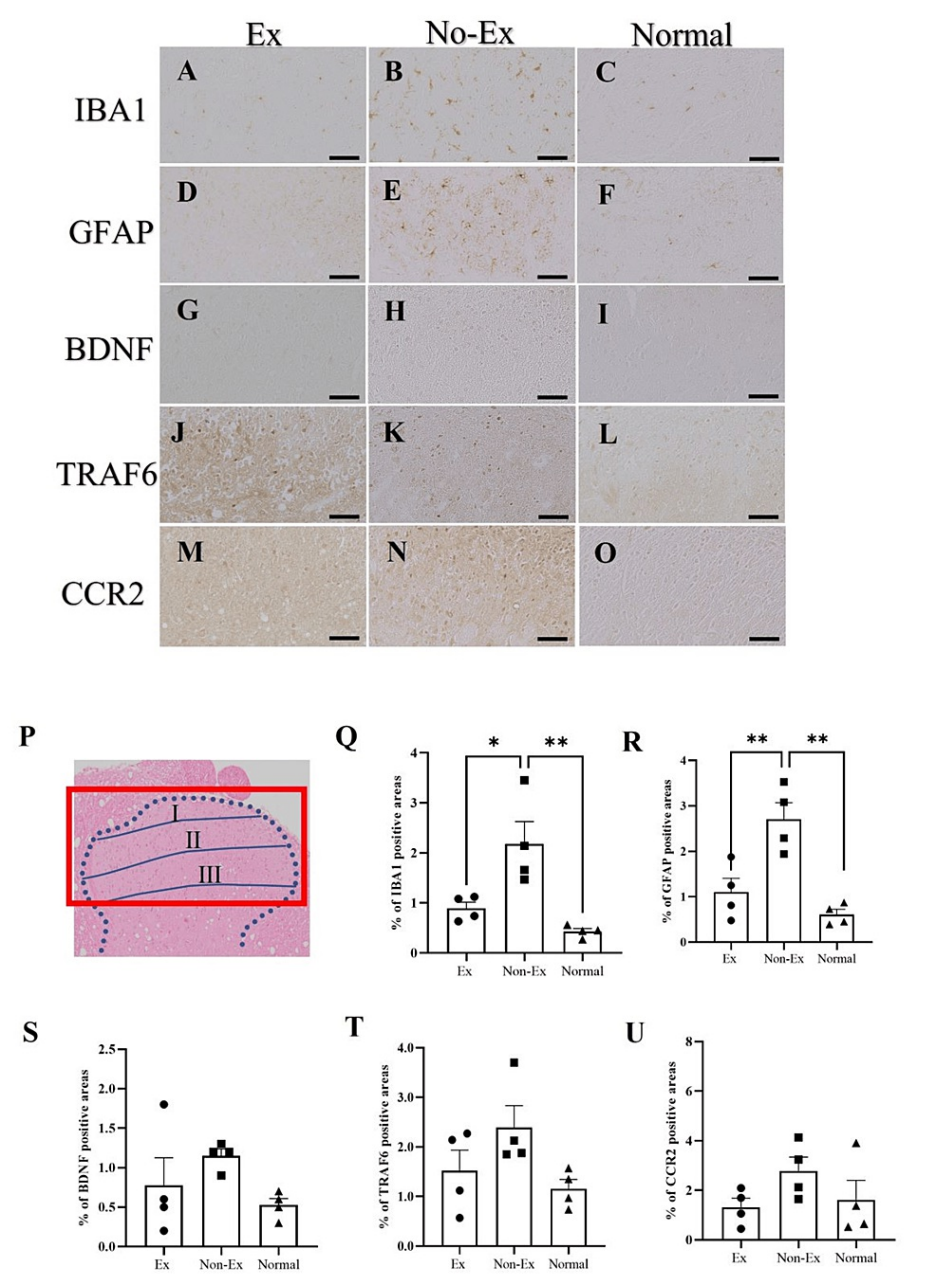
Photographs of immunoreactive DAB staining for IBA1 (A, B, C), GFAP (D, E, F), BDNF (G, H, I), TRAF6 (J, K, L), and CCR2 (M, N, O) in the injured lateral spinal cord dorsal horn at five weeks after CCI. The red square is the quantification site (P). Graphs are a ratio of positive cells for IBA1 (Q), GFAP (R), BDFF (S), TRAF6 (T), and CCR2 (U). The Ex group compared to the No-Ex group, but the difference was insignificant. Data are expressed as mean ± SE. DAB: diaminobenzidine; IBA1: ionized calcium-binding adaptor molecule 1; GFAP: glial fibrillary acidic protein; BDNF: brain-derived neurotrophic factor; CCR2: C-C motif chemokine receptor 2; TRAF6: tumor necrosis factor receptor-associated factor 6

Anti-IBA1-, GFAP-, and BDNF-positive cell area (Figures 2A-2C) ratios at five weeks post-CCI were quantified at the lamina I-III sites in the spinal dorsal horn (Figures 2P-2R).

At five weeks after CCI, the IBA1 positive area ratio was significantly decreased in the Ex group (0.9% ± 0.1%; n = 4) than in the No-Ex group (2.2% ± 0.4%; n = 4) (p < 0.05) (Figures 2A, 2B, 2Q). Regarding GFAP, the Ex group exhibited a significantly (p < 0.01) diminished positive area ratio (1.1% ± 0.3%; n = 4) compared with the respective area ratio of the No-Ex group (2.7% ± 0.4%; n = 4) (Figures 2D, 2E, 2R). BDNF positive area ratio was 0.7% ± 0.4% (n = 4) in the Ex group and 1.1% ± 0.1% (n = 4) in the No-Ex group (Figures 2G, 2H, 2S), which were not significantly different (Figure 2S).

The area ratio for IBA1 positivity in the normal group was 0.4% ± 0.1% (right) (Figures 2C, 2Q, 2F), that of GFAP-positive area ratio was 0.6%±0.2% (right) (Figures 2F, 2R), and that of BDNF-positive area ratio was 0.5% ± 0.1% (right) (Figures 2I, 2S). At five weeks after CCI, the No-Ex group showed a significant increase in IBA1 and GFAP levels compared to the normal group (p < 0.05) (Figures 2B, 2E, 2Q, 2R).

Exercise-induced changes in TRAF6 and CCR2 levels in the dorsal horn of the spinal cord

Similar to IBA1 and GFAP, CCR2 quantification was performed at five weeks post-CCI at the site of the lamina I-III layers in the dorsal horn of the spinal cord (Figure 2P). TRAF6 was quantified at five weeks post-CCI at the same quantification site.

**Figure 3:**
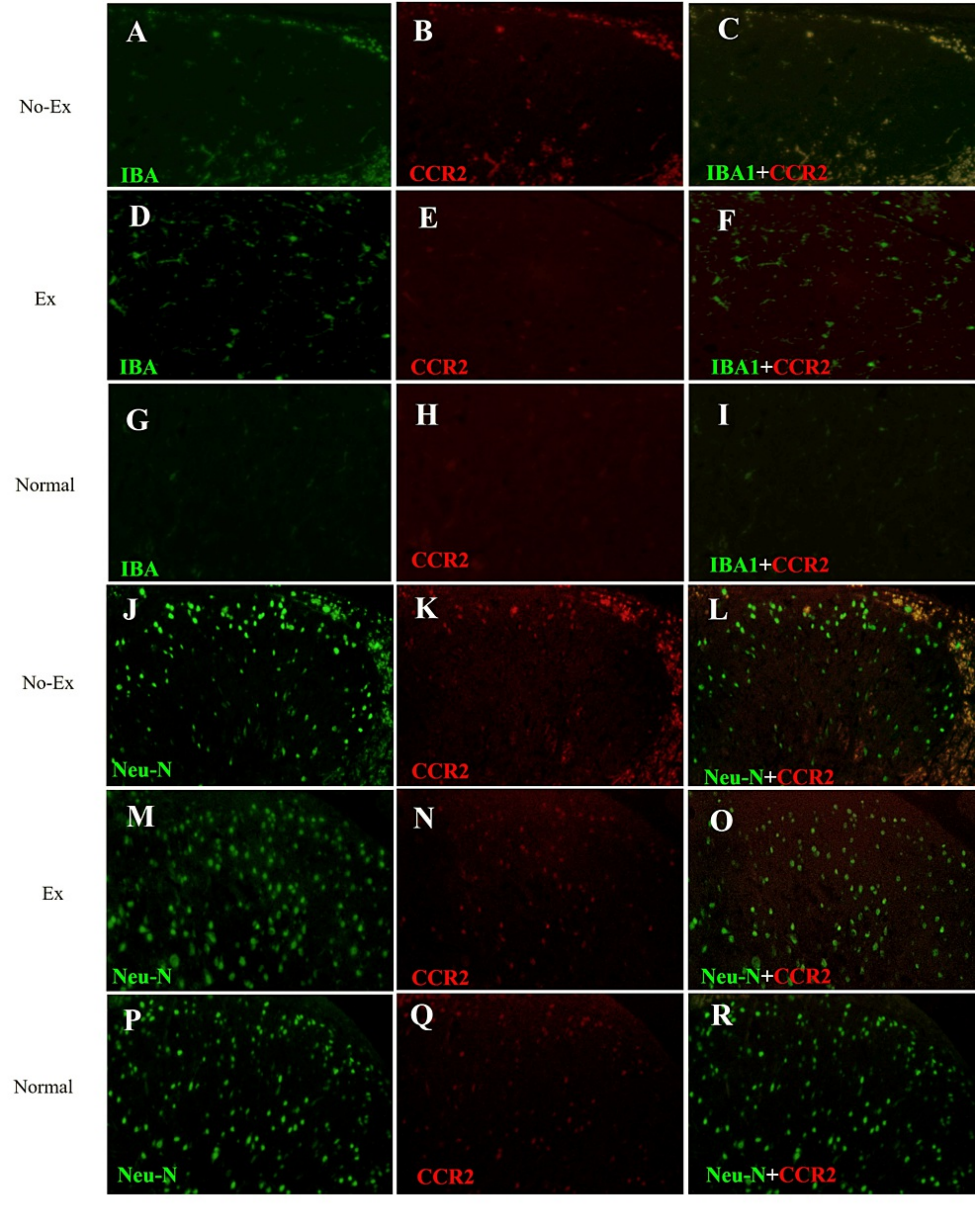
Epidemic reactive fluorescence staining images of IBA1 (A, D, G), Neu-N (J, M, P), CCR2 (B, E, H, K, N, Q), and co-stained images of IBA1 and CCR2 (C, F, I) at five weeks post-CCI in the spinal dorsal horn. In the co-stained images of Neu-N and CCR2 (L, O, R), the co-staining is stronger in the No-Ex group (L), and the co-staining is stronger in the No-Ex group (L) than in the Ex group (O). Neu-N: neuronal nuclear antigen; CCR2: C-C motif chemokine receptor 2; IBA1: ionized calcium-binding adaptor molecule 1; CCR2: C-C motif chemokine receptor 2; Ex: exercise

Five weeks after CCI, TRAF6 positive area ratio was 1.5% ± 0.6% (n = 4) in the Ex group and 2.4% ± 0.4% (n = 4) in the No-Ex group (Figures 3J, 3K, 3T). Moreover, the CCR2 positive area ratio was 1.3% ± 0.4% (n = 4) in the Ex group and 2.8% ± 0.6% (n = 4) in the No-Ex group (Figures 2M, 2N, 2U). At five weeks after CCI, compared with the No-Ex group, the Ex group showed no significant difference for both TRAF6 and CCR2 (Figures 2T, 2U).

The area ratio of TRAF6-positive cells in the normal group was 1.2% ± 0.2% (right) (Figures 2L, 2T). The area ratio of CCR2-positive cells was 1.6% ± 0.8% (right) (Figures 2O, 2U).

The immunoreactive fluorescence staining of the spinal cord dorsal horn (Figure 3) showed strong co-expression of IBA1 and CCR2 in the No-Ex group (Figure 3C) and slight co-expression of IBA1 and CCR2 in the Ex group (Figure 3F). IBA1 and CCR2 co-expression was not observed in the normal group (Figure 3I). Immunoreactive fluorescence staining of neuronal nuclear antigen (Neu-N) and CCR2 (Figures 3J-3R) showed the strongest co-staining as early as five weeks after CCI in the No-Ex group (Figure 3L). At five weeks after CCI, the Ex group also showed co-expression, confirming the continued CCR2 activation in neurons (Figure 3O).

Activation of β-endorphin/met-enkephalin in the gray matter of midbrain aqueduct

**Figure 4:**
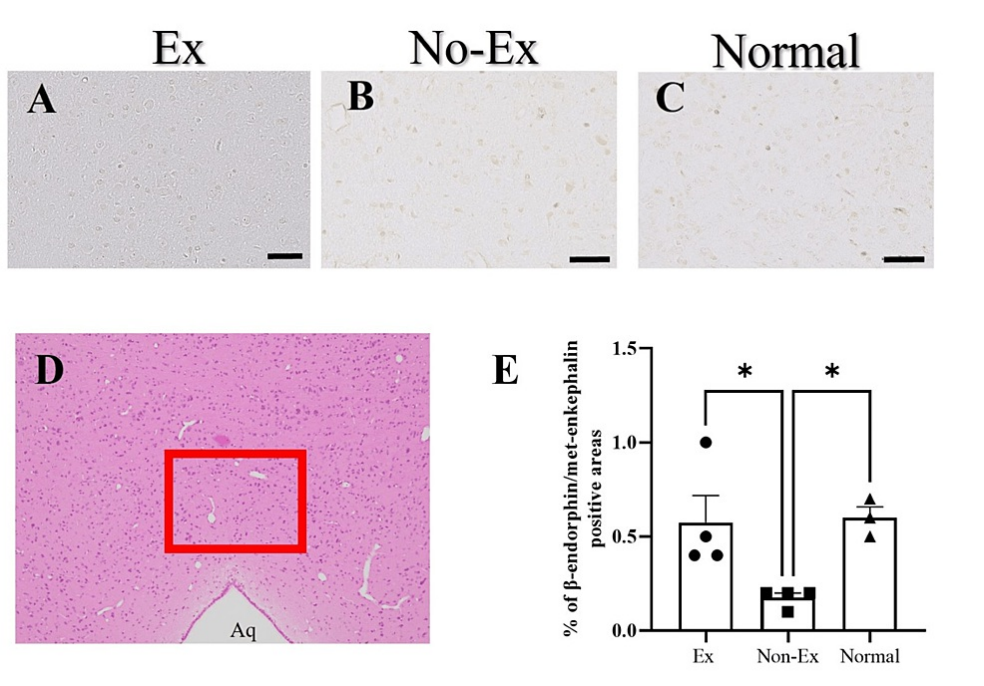
Photographs of immunoreactive DAB staining for β-endorphin/met-enkephalin (A, B, C) in the upper gray matter chamber of the midbrain aqueduct five weeks after CCI. Granular staining is positive cells. The red square is the quantification site (D). The graph shows β-endorphin/met-enkephalin positive cell area ratio (E), with a significant increase in the Ex group compared to the No-Ex group. Data are expressed as mean ± SE. * p < 0.05, ** p < 0.01. Scale bar = 50 μm (all panels). Aq: aqueduct; Ex: exercise; DAB: diaminobenzidine

The area ratio of β-endorphin/met-enkephalin positive cells in the upper gray matter of the midbrain aqueduct was quantified (Figure 4D).

β-endorphin/met-enkephalin was significantly increased in the Ex group (0.6 ± 0.1%; n = 4) than in the No-Ex group (0.2 ± 0.0%; n = 4) at five weeks post-CCI (p < 0.05) (Figures 4A, 4B, 4E). The β-endorphin/met-enkephalin positive cell area ratio in the normal group (n = 4) was 0.5 ± 0.2% (Figures 4C, 4E).

## Discussion

To verify the frequency of exercise intervention for neuropathic pain in this study, the frequency of exercise intervention was minimized to once a week. This helped to verify the best pain-relief effect on neuropathic pain; the mechanism of neuropathic pain in the dorsal horn of the spinal cord in terms of microglia involved in the onset of neuropathic pain; the astrocyte activation associated with the onset and maintenance of neuropathic pain; and the expression of CCR2 and TRAF6. In particular, TRAF6 inhibition, which integrates the inhibition of microglial activation involved in neuropathic pain initiation and the inhibition of CCR2, a chemokine receptor that may be involved in central sensitization in the spinal dorsal horn, is a target for neuropathic pain treatment, although it is often considered in terms of glial cells [17,22].

It is possible that microglia are more involved in the initiation of neuropathic pain, and astrocytes, but not microglial activation, are more involved in their maintenance. We have previously reported that TRAF6 expression in astrocytes is important for maintaining neuropathic pain [17]. Prolonged TRAF6 activation stimulates the JNK/CCL2 pathway in astrocytes, which in turn may contribute to the activation of CCR2, a receptor for CCL2, in neurons and microglia at the primary endings [23]. Other studies have reported that hyperalgesia owing to central sensitization via TRAF6/nuclear factor-κB (NF-κB) was observed in visceral pain models, as knockdown of TRAF6 suppressed colitis-induced activation of NF-κB and increased TNF-α and IL-1β in the spinal cord. This indicates that TRAF6 is always involved in visceral pain and is an upstream event of NF-κB and cytokines, which is consistent with the results of previous studies using neurogenic pain models [9]. In addition, TRAF6 is regulated by cytokines and toll-like receptors and may be involved, via the NF-κB pathway, in the progression of inflammation-related diseases such as diabetes [24]. Forced introduction of miR-125a-5p into astrocytes reduces TRAF6, p-JNK1, and p-JNK2 and suppresses astrocyte activation, rendering miR-125a-5p a possible pharmacological target [25,26]. Elevated TRAF6 levels stimulate neuroinflammation owing to increased TRAF6 binding to K63, as neuropathic pain reduces TRAF6 protein levels in the spinal cord by activating and impairing autophagy [24].

First, regarding the change over time in the 50% withdrawal threshold, the threshold in the Ex group was significantly higher than that in the No-Ex group from the second week after CCI. The Ex group showed a rapid decrease in pain threshold one week after CCI and a significant increase two weeks after CCI compared with that in the No-Ex group. However, the No-Ex group might have temporarily experienced an increased pain threshold because of rest, which could have reduced the pain. This might have prevented a highly significant difference; however, it is significant at three weeks post-CCI. The Ex group then temporarily returned to normal, but there might have been a slight appearance of pain five weeks post-CCI. In our previous study, pain recovery occurred progressively with a five-times-per-week exercise intervention frequency, and the pain returned to normal five weeks after CCI. Our experimental results showed improvement in the reduction of sensory hypersensitivity. However, the weekly exercise intervention tended to be more unstable than the five-times-weekly intervention regarding the pain recovery process. As in a previous study, we focused on three and five weeks post-CCI and examined the changes over time in the 50% withdrawal threshold and intracellular changes in the dorsal horn of the spinal cord.

Regarding the changes five weeks after CCI, the 50% withdrawal threshold in the Ex group was significantly higher than that in the No-Ex group, and the pain threshold was closer to normal in the Ex group, showing even higher significance than that at three weeks after CCI. Conversely, in the dorsal horn of the spinal cord, IBA1 and BDNF showed a significant decrease in the Ex and No-Ex groups, which suppressed disease onset. GFAP, which may be involved in maintenance, also showed significantly decreased expression in the Ex group compared with the No-Ex group, but no significant difference was observed in the expression of TRAF6, which is thought to be expressed in astrocytes, between the Ex and No-Ex groups. In our previous study, we showed the suppression of TRAF6 expression [17]. Thus, TRAF6 suppression may be insufficient. The absence of TRAF6 suppression indicated that TNF-α and IL-1β signaling and the JNK/CCL2 pathway activation were not suppressed. In addition, the expression of CCR2 was downregulated in the Ex and No-Ex groups, but the difference was insignificant. Immunoreactive fluorescence staining of Neu-N and CCR2 also showed stronger co-staining than that of IBA1, although the expression in the Ex group was weaker than that in the No-Ex group five weeks after CCI. It is possible that Neu-N and CCR2 co-expression in the five-week Ex group after CCI might not have achieved the effect of CCR2 suppression on neurons because a slightly higher amount of co-staining was observed when the co-expression was compared between three and five weeks. Immunostaining results also showed the suppression of IBA1 in the Ex group compared with that in the No-Ex group. However, CCR2 was suppressed in microglia but not in TRAF6. This may not have resulted in the inhibition of CCR2 in the primary terminal neurons. CCR2 plays an essential role in the development of persistent neuropathic pain in peripheral macrophages and resident microglia through neuron-macrophage and neuron-microglia interactions [27]. CCR2 is also expressed in astrocytes, similar to CCL2, and CCL2/CCR2 signaling may regulate the maintenance of astrocyte activation through autocrine and/or paracrine processes [28]. CCR2-dependent mechanisms regulate the downstream activation of microglia without directly affecting their initial proliferation, migration, or morphological activation. Cellular elements releasing CCL2, cellular targets expressing CCR2, and infiltration of blood-derived CCR2 cells differ in the spinal dorsal and ventral horns and depend on both the type and the duration of the injury [29]. Five weekly exercise interventions suppressed both CCR2 and TRAF6 at five weeks post-CCI, and pain could be restored to a normal state. However, once-weekly exercise intervention did not result in such suppression and may have resulted in incomplete central sensitization suppression.

In the No-Ex group, endogenous opioid β-endorphin/met-enkephalin showed a significant increase in the brain at five weeks post-CCI compared to that in the Ex and No-Ex groups. Endogenous opioids (β-endorphin, enkephalin, dynorphin) can mimic the effects of morphine and are capable of presynaptic inhibition of transmitter release. Furthermore, the adjacent structures of periaqueductal gray matter, limbic nuclei, and rostral ventral medial medulla with projections to the dorsal horn of the spinal cord constitute a pain control system descending from the brain to the spinal cord; the plasticity of this descending pain control system has been shown to be an integral component of syndromes caused by persistent peripheral disability, emphasizing the need to consider this system as a target for pharmacologic and other types of treatments for chronic pain [30]. In addition, other previous studies have reported that the effects of exercise began to diminish four days after exercise cessation and that endogenous opioid expression decreased rapidly after exercise cessation [15]. In the present study, weekly exercise intervention showed an increase in endogenous opioids. In addition, the 50% pain response threshold returned to its pre-injury state at five weeks post-CCI. Although previous studies have shown that pain begins to diminish four days after exercise cessation, our results suggest that the effect can still be demonstrated with weekly exercise intervention. This implies that neuropathic pain relief is achieved through the activation of the descending pain-inhibitory system.

In the present study, we set the frequency of exercise intervention to a minimum of weekly to test the frequency of exercise intervention that would provide the best pain relief for neuropathic pain. This study aimed to examine the effects of pain relief and the mechanism of neuropathic pain in the dorsal horn of the spinal cord. The above was determined in relation to microglia associated with neuropathic pain initiation and astrocyte activation of neuropathic pain associated with initiation and maintenance of neuropathic pain and expression of CCR2 and TRAF6. At five weeks post-CCI, microglia and astrocytes were suppressed. Glial cells-microglia and astrocytes are the primary factors in neuropathic pain. In the present study, no inhibitory effect was obtained, which may have improved sensory sensitivity. However, CCR2 and TRAF6 were not suppressed, and although pain reduction was possible, the results were not similar to those of our previous study of five-weekly exercise interventions. Therefore, CCR2 and TRAF6 may need to be continuously examined as factors involved in the development and maintenance of neuropathic pain.

## Conclusions

The frequency of the exercise intervention was minimized to weekly to study the effect of pain relief and the mechanism of neuropathic pain in the dorsal horn of the spinal cord. Our results suggest that even if exercise frequency is weekly, it is possible to alleviate neuropathic pain and suppress microglia and GFAP involved in the onset and maintenance of neuropathic pain, respectively.

However, there was no effect of CCR2 suppression, which may be involved in central sensitization, or TRAF6 suppression, which integrates TNF-α and IL-1β signaling. In the present study, neuropathic pain relief was achieved even with minimal exercise intervention.
